# Literature review of leukoencephalopathy with calcifications and cysts and a case report

**DOI:** 10.3389/fnins.2026.1686818

**Published:** 2026-01-16

**Authors:** Chao Guan, Huanting Li, Zechen Wang, Jie Wu, Yunqing Chen, Yugong Feng, Zhenwen Cui, Chen Shen, Pin Guo

**Affiliations:** 1 Qingdao University, Qingdao, China; 2 The Affiliated Hospital of Qingdao University, Qingdao, China

**Keywords:** bevacizumab, Labrune syndrome, LCC, leukoencephalopathy with calcifications and cysts, neurosurgery, SNORD118 mutation, VEGF inhibitor

## Abstract

**Objective:**

To explore the clinical features and treatment approaches for leukoencephalopathy with calcifications and cysts (LCC).

**Methods:**

We retrospectively analyzed a 22-year-old male patient with genetically confirmed LCC admitted to Qingdao University Affiliated Hospital in April 2019. The patient’s clinical presentation, imaging characteristics, treatment course, and outcomes were summarized alongside a comprehensive literature review.

**Results:**

The patient underwent resection of bilateral frontal lobe cysts followed later by resection of a parietal lobe cyst. Postoperative pathology confirmed LCC with calcification and cystic changes. No severe postoperative complications occurred. Follow-up imaging demonstrated gradual cyst regression and symptom resolution. Genetic testing identified heterozygous SNORD118 variants (n.3C>T and n.74G>A).

**Conclusion:**

Surgical resection is an effective treatment for LCC cysts causing significant mass effect or neurological deficits, requiring regular follow-up. For smaller, asymptomatic cysts without mass effect, treatment with VEGF inhibitors (e.g., bevacizumab) may be beneficial. Management should be individualized.

## 1 Introduction

**Figure 1:**
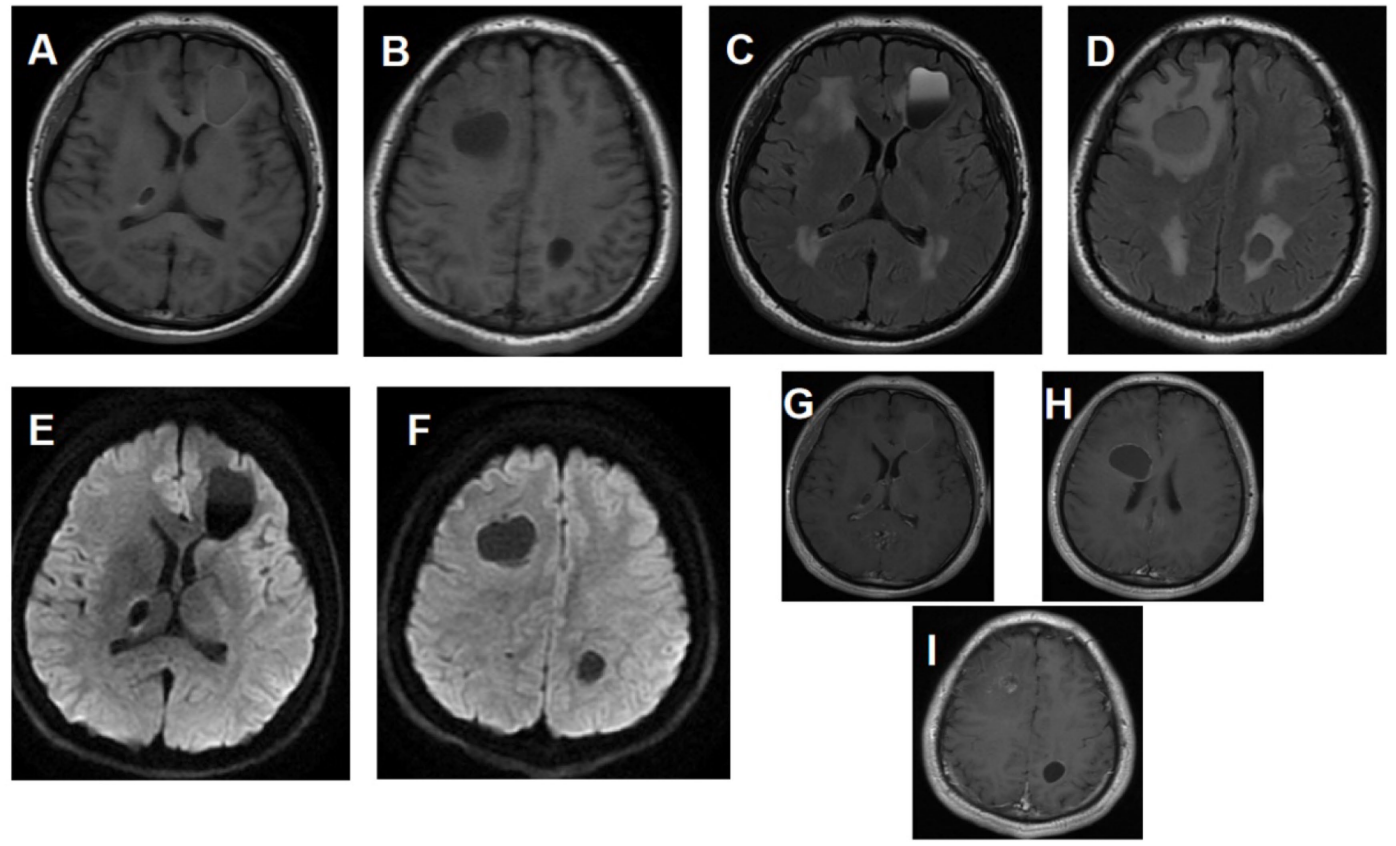
Cranial MRI and enhancement at the time of patient’s visit in April 2019. **(A)** MRI T1 shows fluid signals in the right frontal lobe and left parietal lobe, accompanied by edema of surrounding brain tissue. **(B)** MRI T1 reveals mixed signal shadows in the left frontal lobe and low-density shadows in the right thalamus. **(C,D)** MRI T2 shows cysts in the right frontal lobe and left parietal lobe, nodular low signal shadows in the right thalamus, and solid cysts in the left frontal lobe. **(E,F)** MRI diffusion-weighted imaging shows cystic lesions with restricted diffusion. **(G–I)** MRI enhancement shows multiple white matter lesions, with no enhancement observed in the cysts.

**Figure 2:**
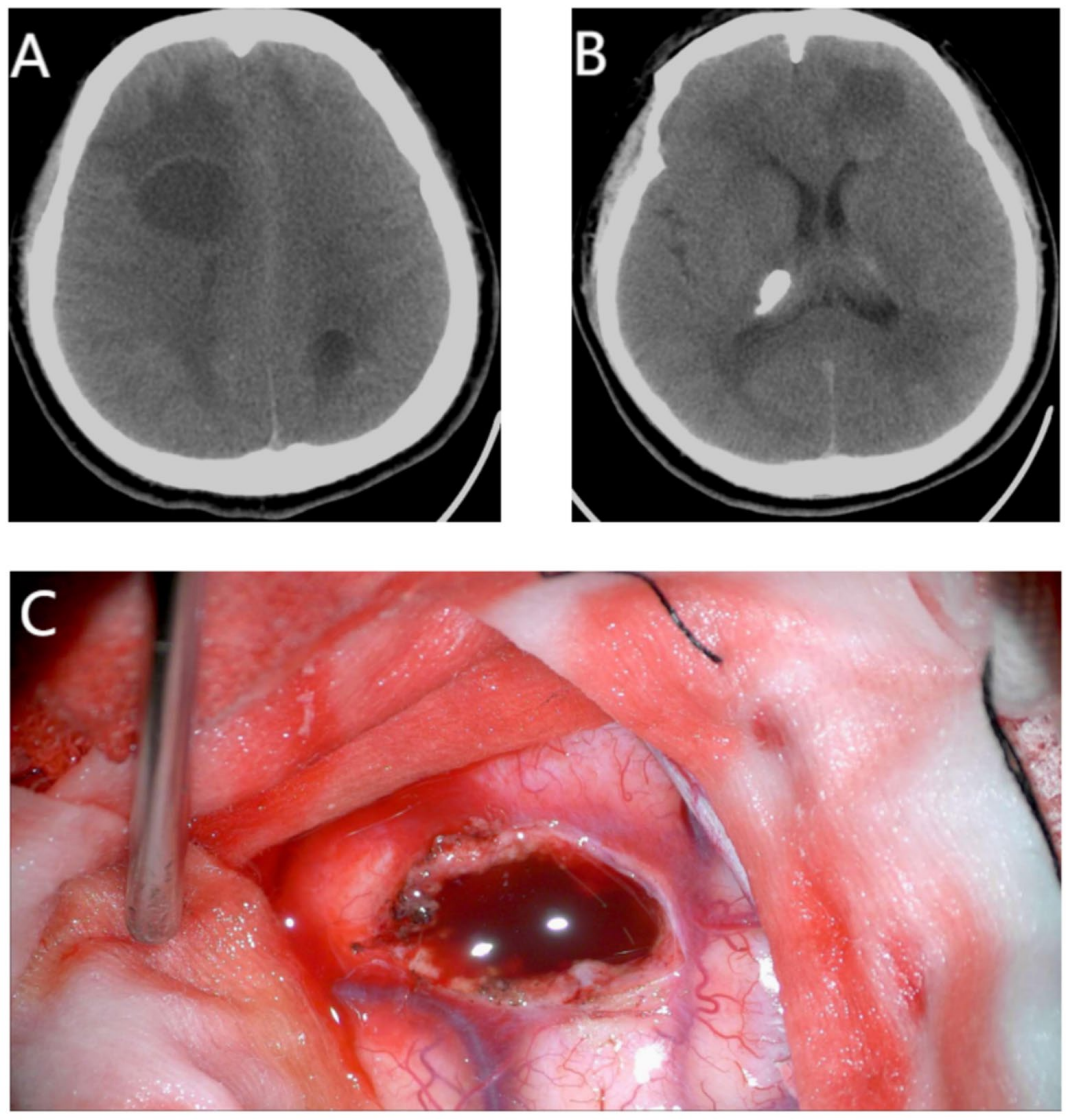
Preoperative cranial CT and intraoperative right frontal lobe cystic lesion of patient in April 2019. **(A,B)** Preoperative cranial CT showed cysts in bilateral frontal lobe and left parietal lobe, calcification of right thalamus. **(C)** Lesions in right frontal lobe with cyst fluid.

**Figure 3:**
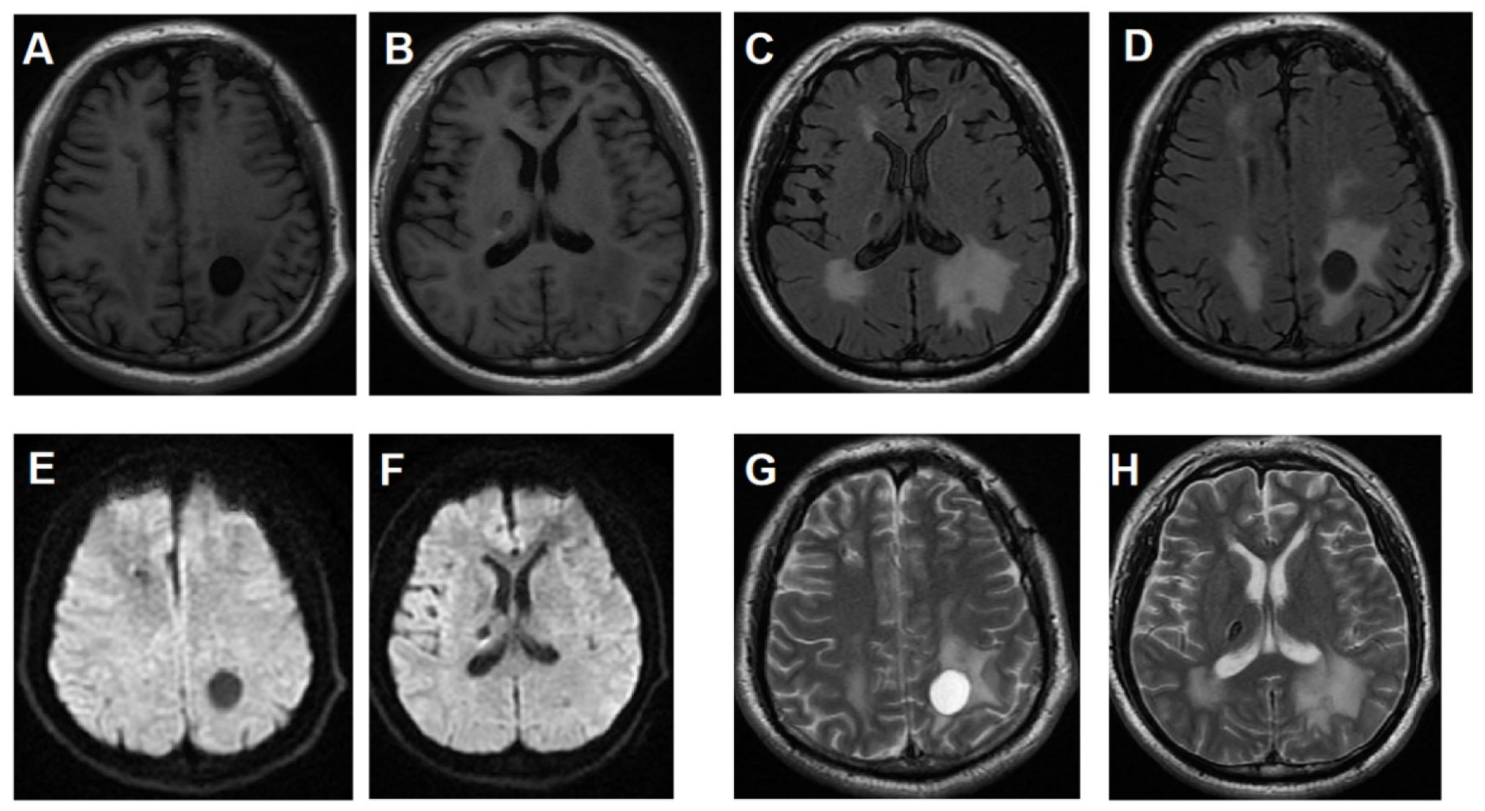
Cranial MRI review of patient 2019 April two years after surgery. **(A,B)** MRI T1 **(C,D)** MRI T2 **(E,F)** MRI diffusion weighted imaging **(G,H)** MRI AXIFSET2WI2. Bilateral frontal lobe cysts disappeared, the volume of left occipital lobe cyst increased compared with before, and the calcification of right thalamus remained unchanged.

**Figure 4:**
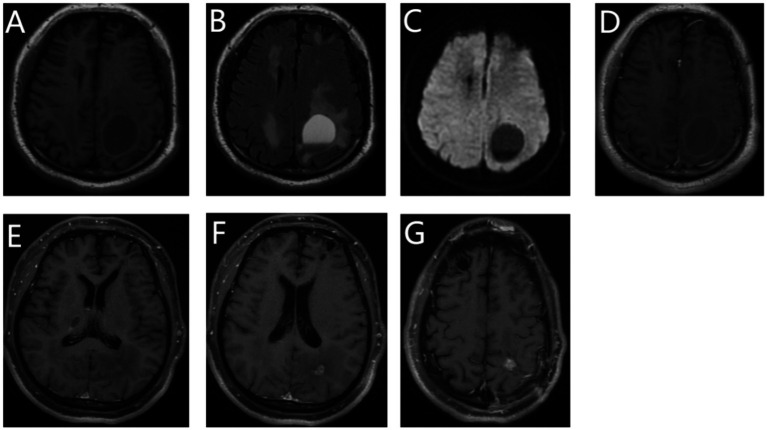
Preoperative cranial MRI of patient 2024 February, and postoperative cranial MRI enhancement 4 months after surgery. **(A–D)** Preoperative cranial MRI cyst volume increased significantly in February 2024. **(E–G)** Cranial MRI enhancement in July 2024: cyst disappeared, small nodules were seen in the surgical area.

Leukoencephalopathy with calcifications and cysts (LCC), also known as Labrune syndrome, is a rare disorder characterized by diffuse cerebral microangiopathy leading to myelin pallor, axon loss, white matter rarefaction, intracranial calcifications, and parenchymal cyst formation. First described by Labrune et al. (1996), fewer than 100 detailed cases had been reported in databases (including CNKI, Wanfang, PubMed, Web of Science, Embase, CBM, VIP) as of July 2022 (Li et al., 2023). This report details a gene-confirmed case of LCC, in which the patient was a 22-year-old male. In early April 2019, the male experienced diplopia, headache, nausea and vomiting. The symptoms indicated intracranial hypertension, so he underwent head MRI and CT examinations. Imaging results showed multiple intracranial white matter lesions, bilateral frontal and left parietal cysts with peripheral edema, and calcification of the right thalamus (Figures 1, 2A,B). The initial diagnosis was LCC. In mid-April 2019, the patient underwent microsurgical neurosurgery at our hospital, performing bilateral frontal lobe cystectomy through cortical fenestration (Figure 2C). One year after the operation, a reexamination of the head MRI showed that only nodular shadows remained in the bilateral frontal lobe cysts, but the left parietal lobe cyst was enlarged (Figure 3). In February 2024, the patient suddenly experienced right limb convulsions accompanied by loss of consciousness. Re-examination of head MRI revealed that the left parietal cyst was significantly enlarged, with obvious peripheral edema (Figures 4A–D). A cortical fenestration parietal cyst resection surgery was performed. Four months after the operation, re-examination showed no recurrence of the parietal cyst (Figures 4E–G). At the end of 2024, genetic testing of the patient and his family revealed a SNORD118 mutation, and he was ultimately diagnosed with hereditary LCC. We present the clinical, imaging, pathological, and genetic findings, combined with a literature review, to enhance understanding of LCC diagnosis and management.

## 2 Case report

### 2.1 Clinical presentation and imaging

A 22-year-old male presented to Qingdao University Affiliated Hospital on April 22, 2019, with a 20-day history of right eye diplopia, headache, nausea, and vomiting. Symptoms began without obvious precipitant. Initial supportive care at a local hospital provided no improvement. Cranial CT revealed multiple intracranial lesions. Subsequent cranial MRI and contrast-enhanced MRI at our institution demonstrated multiple brain lesions, suggestive of LCC. He was admitted to the neurosurgery department for “intracranial mass lesions.”

Past medical history was unremarkable. Admission examination revealed: clear consciousness; fluent speech; normal hearing; symmetrical facial features; midline tongue protrusion; bilateral pupils 3.0 mm, equal, reactive to light; normal extraocular movements except right eye diplopia; normal visual fields; normal superficial sensation; normal limb muscle strength and tone; negative Babinski sign; and no nuchal rigidity.

#### 2.1.1 Brain CT

Calcifications in the right basal ganglia and bilateral thalamus. Cystic hypodensities with relatively defined borders were seen in the deep white matter of the bilateral frontoparietal lobes, surrounded by vasogenic edema. A fluid level was noted within the left frontal lobe cyst. No significant supratentorial ventricular enlargement or sulcal widening. Midline structures were midline.

#### 2.1.2 Brain MRI

Multiple cysts in the frontal lobes and left parietal lobe showing long T1, mixed long/short T2 signals, FLAIR mixed hyper/hypointensity, and DWI hypointensity. A nodular hypointense lesion was present in the right thalamus. Patchy slightly prolonged T1 and T2 signal abnormalities were seen in the bilateral centrum semiovale and periventricular white matter, showing FLAIR hyperintensity and DWI isointensity. Mild supratentorial ventricular and sulcal prominence was noted. Midline structures were midline.

#### 2.1.3 Contrast-enhanced MRI

Cyst walls in the frontal and left parietal lobes demonstrated enhancement. The right thalamic nodule showed mild marginal enhancement. The white matter abnormalities showed no enhancement.

### 2.2 Diagnostic evaluation

The patient was admitted with blurred vision as the initial symptom and presented with cranial imaging data. Upon admission, further cranial MRI with contrast enhancement was performed. Cranial CT revealed bilateral frontal lobe and left parietal lobe cysts with cystic fluid, right thalamic calcification, and significant pericystic cerebral edema in both frontal lobes, accompanied by left shift of midline structures. An ophthalmology consultation was arranged for diagnostic assistance, including visual acuity, visual field, and fundus examinations, which identified bilateral optic disc edema. Based on imaging findings and clinical manifestations, the patient’s blurred vision was attributed to increased intracranial pressure. Consequently, a surgical resection of the bilateral frontal lobe cysts with significant space-occupying effects was planned. Postoperatively, antiepileptic therapy and close follow-up were administered to monitor changes in the surgical site and occipital lobe cysts.

### 2.3 Surgical intervention

Under general anesthesia and supine positioning with head fixation, bilateral frontal lesions were targeted. A frontal cross-midline arcuate incision was made. Following craniotomy (right frontal: 4 × 4 cm; left frontal: 3 × 4 cm), microscopic exploration revealed cystic lesions containing brownish-red fluid and light red, nodular cyst walls. The cysts were microsurgically excised along their walls. Hemostasis was achieved meticulously. The surgical sites were irrigated copiously with normal saline. Duroplasty was performed using artificial dura. Drains were placed, and the incision was closed.

Histopathology confirmed leukoencephalopathy with calcification and cystic changes. Immunohistochemistry (2019 Resection): GFAP (+), IDH1 (+), ATRX (+), Olig-2 (+), S100 (+), NF (+), CD68 (−), CD138 (−), CD34 (Vascular+), CD3 (T cell+), CD20 (B cell+), NeuN (neuron+).

### 2.4 Follow-up results

**Figure 5:**
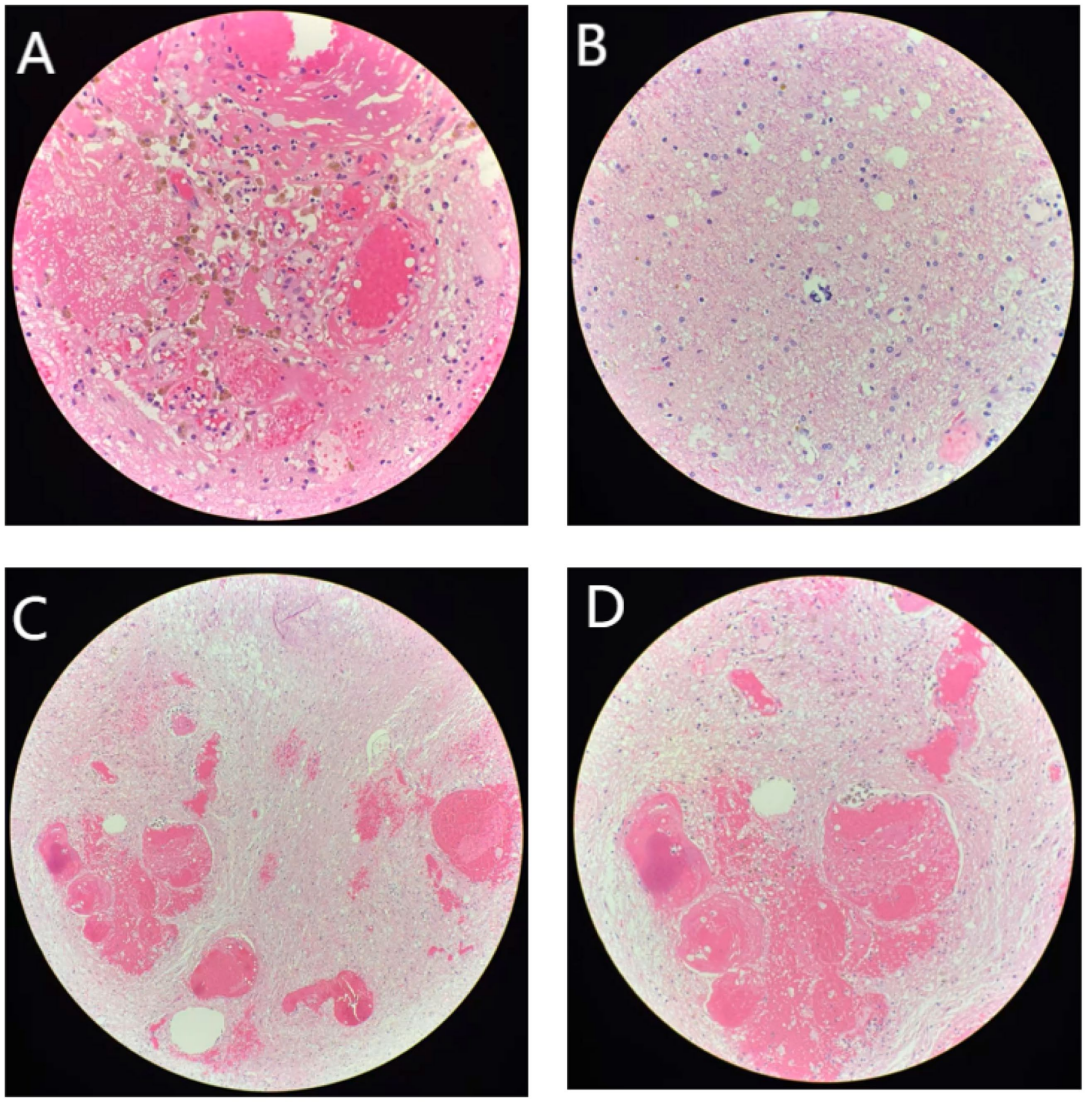
Postoperative pathological tissue section of patient in February 2024. **(A)** Hemorrhage (×40). **(B)** Calcification (×40). **(C)** Hemorrhage with calcification (×100). **(D)** Hemorrhage with calcification (×200).

**Figure 6:**
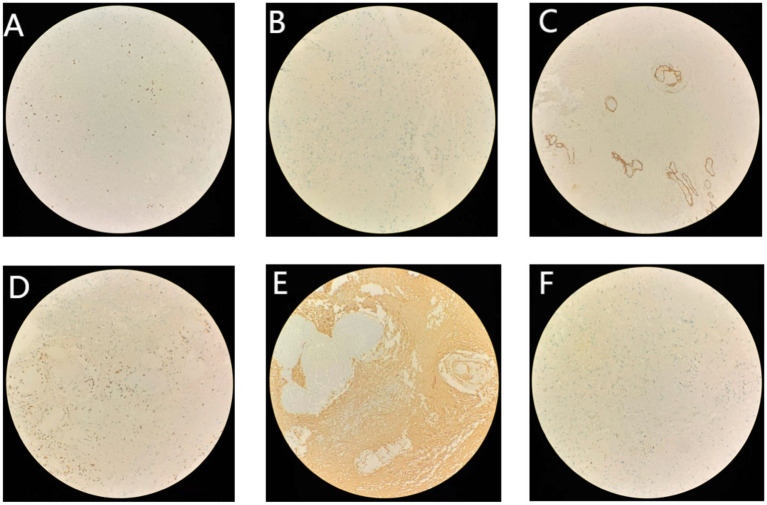
Immunohistochemistry. **(A)** CD3^+^ (×200). **(B)** CD20^−^ (×200). **(C)** CD34^−^ (×200). **(D)** CD68^+^ (×200). **(E)** GFAP^+^ (×200). **(F)** Ki67^+^ (×200).

At two-years postoperative follow-up, gradual reduction of the frontal lobe lesion was observed. The bilateral frontal lobe cysts, initially shown in Figure 1 (left frontal lobe: 34.52 × 27.62 × 30 mm; right frontal lobe: 22.36 × 30.25 × 20 mm), had transformed into a single nodular shadow in both frontal lobes as depicted in Figure 3. However, the left parietal lobe lesion exhibited slow enlargement, increasing from (17.10 × 16.11 × 20 mm) in Figure 1 to (17.47 × 19.52 × 20 mm) in Figure 3. During the follow-up period, the patient underwent surgical treatment for orbital fracture caused by trauma in September 2021, without undergoing cranial imaging examinations. It remains uncertain whether this traumatic event accelerated the growth of the cranial cyst. In February 2024, the patient was readmitted for surgical intervention due to limb convulsions. Preoperative imaging revealed a parietal lobe cyst measuring (33.28 × 38.83 × 35 mm). Four months postoperatively, the follow-up imaging (Figure 4) showed only a minimal nodular shadow of the parietal lobe cyst. Postoperative pathological examination revealed leukoencephalopathy with calcification and cystic changes. Immunohistochemical findings included GFAP (+), Olig-2 (+), CD68 (histiocytic positive), S100 (+), IDH1 (−), NF (+), NeuN (−), ATRX (+), CD38 (plasma cell positive), CD3 (T cell positive), CD20 (−), CD34 (−), and Ki-67 (+, approximately 1%). Special staining: Congo red (−). In November 2024, the patient underwent genetic testing, which revealed the following findings: SNORD118; NR-033249.1: heterozygous variants n.3C>T and n.74G>A (see Figures 5, 6).

## 3 Discussion

LCC is a rare autosomal recessive disorder. Jenkinson et al. (2016) identified biallelic mutations in SNORD118 (a non-protein-coding small nucleolar RNA gene on chromosome 17p13.1) as the cause. LCC represents the first human disease attributed to mutations in a gene encoding a C/D-box snoRNA. However, rare SNORD118 mutation-negative cases exist (Rohatgi et al., 2024), suggesting potential unidentified genetic causes. In this case, genetic testing revealed a heterozygous variant in the SNORD118 gene: the N.3C>T and n.74G>A mutations at the NR-033249.1 locus, which were inherited from the parents (the father showed the N.3C>T mutation at NR-033249.1, and the mother showed the n.74G>A mutation at NR-033249.1). According to the ACMG guidelines, this variant was classified as a nonsensical mutation (PM2 + PM3-supporting). Although there have been reports of this variant, the exact cause of this disease remains unclear (Jenkinson et al., 2016; Jin et al., 2021; Crow et al., 2021; Wang et al., 2022).

Labrune’s et al. (1996) initial description involved progressive neurological decline in children with leukoencephalopathy, calcifications, and cysts, without extra-neurological involvement. Subsequent reports described patients with retinal telangiectasia alongside neurological features, suggesting overlap between LCC and Coats plus (CP) syndrome, termed cerebroretinal microangiopathy with calcifications and cysts (CRMCC) (Linnankivi et al., 2006). However, Anderson et al. (2012) discovered that CP is caused by CTC1 mutations and is a multisystem disorder featuring retinal telangiectasia/exudates, intracranial calcifications/cysts/leukodystrophy, osteopenia, poor fracture healing, and gastrointestinal bleeding/portal hypertension. Crucially, CTC1 mutations are absent in purely neurological LCC cases (Anderson et al., 2012; Livingston et al., 2014). Livingston et al. (2014) confirmed LCC as a distinct, purely neurological entity characterized neuroradiologically by CP features but genetically by SNORD118 mutations and absence of CTC1 mutations or systemic involvement. Therefore, classifying LCC under CRMCC is inappropriate. Common neuroimaging features of LCC include bilateral diffuse white matter T2 hyperintensity suggestive of white matter lesions. Brain calcifications typically present as asymmetrically scattered lesions in the white matter or deep gray nuclei, manifesting as small punctate foci or larger confluent areas. Cysts within the brain parenchyma are also unevenly distributed, most frequently located supratentorially, with wall enhancement and mass effect being characteristic features. Diffusion-weighted imaging reveals increased water content in abnormal white matter. Collectively, these findings indicate that blood–brain barrier disruption and white matter edema are hallmark features of LCC rather than demyelinating lesions, consistent with the microangiopathic pathogenesis. Although imaging differential diagnoses may include various parasitic infections or metabolic disorders, the triad of diffuse white matter T2 hyperintensity, brain calcifications, and parenchymal cysts (without retinal or extracranial manifestations) should prompt the diagnosis of LCC (Paff et al., 2022).

LCC’s hallmark pathology is diffuse microangiopathy causing progressive vascular occlusion, leading to necrosis, dystrophic calcification, white matter rarefaction, and cyst formation. In immunohistochemical staining, immunostaining for neurofilament (NF) protein revealed axonal loss in the affected white matter. Astrocytes (GFAP) exhibited senescent morphology with larger vacuolar septa in their cytoplasm. Immunomarkers such as CD4 and CD20 showed reduced expression, as did Ki67 staining indicating diminished cell proliferation. It remains unclear why the variant SNORD118 causes this disease that can induce neurological disorders and significantly disrupt the cerebrovascular system, nor is it certain whether this variant may affect other organ systems (Helman et al., 2021). The pathological and immunohistochemical results of the two surgeries in this case supported the diagnosis of LCC.

### 3.1 Medical management

Anti-VEGF therapy, specifically bevacizumab, shows promise. Fay et al. (2017) reported clinical (improved mobility, bradykinesia) and radiological (reduced cysts/white matter lesions) improvement in an 18-year-old patient receiving bevacizumab (5 mg/kg every 2 weeks for >1 year). Scaffei et al. (2023) documented stabilization in a 10-year-old patient after cyst enlargement post-drainage. Larger controlled trials are needed to define optimal dosing, timing, and consequences of discontinuation.

### 3.2 Surgical management

Novegno et al. (2024) reviewed neurosurgical approaches (drainage, aspiration, resection). Recurrence requiring re-intervention (either same cyst or new cyst) occurred in approximately 50% of cases, more frequently in pediatric patients. Recurrences typically manifest weeks to 2 years postoperatively, while new cysts can develop over 15 years. Aggressive resection is feasible, but potential neurological deficits must be weighed against radiological benefits, particularly in asymptomatic cases. Complete cyst removal is not always necessary, as aspiration alone can yield durable results. Surgical planning must be individualized, prioritizing neurological preservation. Given the disease’s unpredictable course, long-term clinical and radiological follow-up (minimum annual imaging) is recommended, even in stable patients (Novegno et al., 2024). In this case, both surgeries employed the cortical fenestration approach for cyst resection. The first procedure was initiated due to intracranial hypertension (ICH), with significant postoperative relief of intracranial pressure. The patient was prescribed oxcarbazepine for seizure prophylaxis, and no epileptic seizures were observed for three months postoperatively, leading to discontinuation of the medication. The second surgery was performed for limb convulsions accompanied by loss of consciousness, with only one such episode occurring. Imaging findings indicated that the edema of the parietal lobe cyst had extensively involved the frontal lobe, confirming the diagnosis of epileptic seizures. The patient has not experienced recurrence since the surgery. Follow-up imaging confirmed no recurrence of the intracranial cyst, demonstrating the substantial benefits of the surgical approach chosen for this patient.

## 4 Conclusion

LCC is a rare disease, and cranial imaging of the case we shared showed calcification, cysts, and white matter degeneration, and fundus examination showed optic disc edema without other fundus lesions and other systemic involvement. Pathological examination suggests senescence and microangiopathy of nerve cells. Combined with SNORD118 gene mutation testing, the diagnosis of LCC can be finally confirmed. Bevacizumab is a promising medical therapy that deserves further clinical trials to develop guidelines. Surgical intervention (excision or aspiration) is effective for symptomatic cysts that cause a lump effect, but recurrence and new cyst formation require long-term monitoring. Comprehensive treatment strategies for LCC require further investigation through larger case series.

## Data Availability

The original contributions presented in the study are included in the article/supplementary material, further inquiries can be directed to the corresponding author.
